# Launching the Living Alone With Cognitive Impairment (LACI) Project: Bridging Research and Policy

**DOI:** 10.1093/geroni/igab046.269

**Published:** 2021-12-17

**Authors:** Elena Portacolone, Julene Johnson, Jacqueline Torres, M Maria Glymour, Sahru Keiser, Thi Tran

**Affiliations:** 1 UCSF, University California San Francisco, California, United States; 2 University California San Francisco, San Francisco, California, United States; 3 University of California, San Francisco, San Francisco, California, United States; 4 UCSF/ San Francisco, UCSF Institute of Health and Aging/ San Francisco, California, United States

## Abstract

The Living Alone with Cognitive Impairment (LACI) Project bridges research and policy to develop policy recommendations to address the needs of people living alone with cognitive impairment (PLACI) through new expansions of long-term services and supports. There are an estimated 4.3 million PLACI in the United States. Access to formal LTSS is critical to them because they lack cohabitants to assist with activities of daily living and navigating LTSS, especially during the COVID-19 pandemic. To bridge research with policy, seventeen Policy Advisory Group (PAG) members were recruited, including representatives from state and local government, and LTSS policy experts. Between November 2020-January 2021, a total of 17 individual meetings were conducted with PAG members and one webinar convening of the group. The PAG identified preliminary recommendations in three areas, including: 1) important areas of inquiry for qualitative and quantitative research, 2) best practices for addressing equity across diverse racial/ethnic minority groups, and 3) preliminary policy recommendations that leverage existing innovations. The LACI Project team is actively incorporating the PAG feedback by: a) modifying research questions for the quantitative and qualitative research, b) convening a diverse Community Advisory Group, and c) crafting preliminary policy recommendations based on PAG input. To conclude, engaging the expertise of the PAG to develop policy recommendations to increase LTSS for PLACI is a promising method of bridging research and policy. The engagement of policy experts ensures that fore-coming research is designed to address the most important policy gaps and all policy recommendations are actionable and timely.

